# Identifying the need for a UK colorectal cancer screening programme for patients with cystic fibrosis (CF): 10-year retrospective review of colonoscopy and colorectal cancer outcomes at a single CF centre

**DOI:** 10.1136/bmjgast-2023-001178

**Published:** 2023-07-31

**Authors:** Karuna Sapru, Peter Barry, Andrew Jones, John Walmsley, Javaid Iqbal, Dipesh H Vasant

**Affiliations:** 1 Adult Cystic Fibrosis Centre, Wythenshawe Hospital, Manchester University NHS Foundation Trust, Manchester, UK; 2 Division of Immunology, Immunity to Infection & Respiratory Medicine, University of Manchester, Manchester, UK; 3 Gastroenterology Department, Wythenshawe Hospital, Manchester University NHS Foundation Trust, Manchester, UK; 4 Division of Diabetes, Endocrinology and Gastroenterology, University of Manchester, Manchester, UK

**Keywords:** colonoscopy, colorectal cancer, cystic fibrosis, screening

## Abstract

**Objective:**

Patients with cystic fibrosis (pwCF) have a high incidence of early colorectal cancer (CRC). In the absence of a UK CRC screening programme for pwCF, we evaluated the utility and outcomes of colonoscopy and CRC at a large UK CF centre.

**Design:**

In a retrospective study of colonoscopy and CRC outcomes between 2010 and 2020 in pwCF aged≥30 years at a large CF centre, data were collected on colonoscopy indications and findings, polyp detection rates, bowel preparation regimens and outcomes, colonoscopy completion rates, and patient outcomes.

**Results:**

We identified 361 pwCF aged ≥30 years, of whom 135 were ≥40 years old. In the absence of a UK CRC screening guideline only 33 (9%)/361 pwCF aged ≥30 years (mean age: 44.8±11.0 years) had a colonoscopy between 2010 and 2020. Colonoscopy completion rate was 94.9%, with a 33% polyp detection rate, 93.8% of the polyps retrieved were premalignant. During the study period no patients developed postcolonoscopy CRC. However, of the patients aged ≥40 years who did not have a colonoscopy (111/135, 82.2%), four (3.6%) patients developed CRC and three pwCF died from complications of CRC.

**Conclusion:**

In this 10-year experience from a large CF centre, colonoscopy uptake for symptomatic indications was low, yet of high yield for premalignant lesions in pwCF >40 years. These data highlight the risk of potentially preventable, early CRC, and therefore support the need for prospective, large-scale nationwide studies which may inform the need for UK CRC screening guidelines for pwCF.

**WHAT IS ALREADY KNOWN ON THIS TOPIC**

- Patients with cystic fibrosis (pwCF) are at higher risk of early colorectal cancer (CRC) compared with age-matched individuals without cystic fibrosis (CF) and are therefore likely to benefit from bowel cancer screening at a younger age compared with the general population.

**WHAT THIS STUDY ADDS**

- In the absence of a national screening CRC screening programme for pwCF in the UK, only 17.8% of those over ≥40 years had a colonoscopy over a 10-year period at a large CF centre, with a 3.6% potentially preventable CRC rate.

**HOW THIS STUDY MIGHT AFFECT RESEARCH, PRACTICE OR POLICY**

- In an era where the life expectancy of pwCF is increasing, this study raises awareness of increased CRC risk in pwCF above the age of 40. This is important in countries where no current national CRC screening guidelines exist for pwCF. The study therefore highlights the need for future large-scale studies to evaluate the national picture as a prelude to developing UK CRC screening programmes for pwCF.

## Introduction

Cystic fibrosis (CF) is the most common life-limiting autosomal recessive genetic disorder in Caucasians in the UK. It is a multisystem disease resulting from mutations in the gene which encodes CF transmembrane conductance regulator (CFTR) protein; a complex chloride channel involved in producing sweat, mucous, tears and digestive enzymes. The median age of survival in patients with cystic fibrosis (pwCF) has increased. According to the CF registry report half of pwCF born today are predicted to live to at least 53.3 years,^1^ as a result of improvements in diagnostic and therapeutic options, and is likely to rise further with recent availability of treatments targeting CFTR mutations. There is a growing population of adults with CF and it is estimated that 36% of adults with CF will be over 40 years by 2030.^2^

The risk of developing colorectal cancer (CRC) in pwCF is 5–10-fold higher compared with age-matched individuals without CF, and the risk of CRC increases up to 30 times post solid organ transplant.^3^ Non-specific gastrointestinal symptoms are common in CF and make the diagnosis of CRC challenging. This increased risk of CRC has led to the development of the North American CF CRC screening recommendations, advising clinicians to commence endoscopic screening by the age of 40 years and even earlier at aged 30 years if the patient has had a solid organ transplant.^4^

The mechanisms responsible for increased risk of CRC in CF are unclear. The CFTR gene has been shown to be a tumour suppressor gene in animal studies, where loss of CFTR is associated with intestinal tumour formation. CFTR dysfunction or deficiency may potentially contribute to disruption of intestinal stem cell homeostasis, intestinal barrier integrity, intestinal dysbiosis and inflammation.^5^ Some studies suggest the increased risk for CRC may also extend to heterozygous carriers of CFTR mutations.^6^ In patients with sporadic CRC, low expression of CFTR has been associated with poor survival.^7^ In view of the increased risk of CRC in pwCF and in the absence of a national UK screening programme, we aimed to evaluate the current utility and diagnostic yield of colonoscopy, the incidence of CRC and the proportion of patients that would potentially benefit from CRC screening programme at a CF centre.

## Methods

We carried out a retrospective cohort study of pwCF attending the Manchester Adult Cystic Fibrosis Centre (MACFC) between 2010 and 2020. Data were collected from a prospectively maintained Microsoft excel database of all patients with a diagnosis of CF, based on genetic mutation and sweat chloride results. Patients were included if they were aged 30 years or older. For those who had undergone a colonoscopy within the 10-year study period, data were collected on patient symptomatology and family history from outpatient clinic letters. Colonoscopy reports using Unisoft Medical Systems endoscopy software reporting tool were accessed to collect data on indication for procedure, colonoscopy findings provided by the endoscopist including number of polyps identified and removed, bowel preparation used, Boston Bowel Prep Score (BBPS) or equivalent description. BBPS is used by endoscopists at our centre to classify adequacy of the bowel prep given for each colonoscopy. A score was given from 0 to 9; with 0–4 being ‘inadequate’, 5–6 being ‘satisfactory’, 7–8 being ‘good’ and 9 being ‘excellent’. Endoscopists could alternatively specify the above terms in their report, in which cases a score would be allocated according to their description. Additional data obtained from colonoscopy reports included if diagnostic views were achieved during colonoscopy and whether colonoscopy was completed. Polyp histology findings were obtained from pathology reports through electronic patient records. Premalignant polyps were defined as per British Society of Gastroenterology/Association of Coloproctology of Great Britain and Ireland/Public Health England postpolypectomy and post-CRC resection surveillance guidelines. The term includes both serrated polyps (excluding diminutive (1–5 mm) rectal hyperplastic polyps) and adenomatous polyps. It does not include other polyps such as post-inflammatory polyps.^8^

Patient outcomes post colonoscopy were obtained via clinical case notes. Statistical analysis was carried out using IBM SPSS Statistics V.25. For all analyses p value <0.05 was considered statistically significant. As this was a retrospective service evaluation of clinical practice, ethics approval was not required. Institutional authorisation to hold a prospective patient database for use for quality improvement was obtained. (Registration number HL 091).

## Results

A total of 709 patients attended MACFC between 2010 and 2020. Of these we identified 361 (51%) patients aged ≥30 years, of which 135 were 40 years and above. A total of 33 (9%)/361 pwCF aged ≥30 years (mean age: 44.8±11.0 years) underwent 39 colonoscopies between 2010 and 2020. Two patients had three procedures each. Of the 33 pwCF who underwent an index colonoscopy, 24 (18%) patients were aged ≥40 years, of which only one patient underwent a colonoscopy for screening due to a family history of CRC. The remaining 23 patients were referred due to gastrointestinal symptoms, which included altered bowel habit, constipation, diarrhoea, weight loss and per rectal bleeding. Of the patients aged 30–39 years, 9 (4%)/226 patients went on to have a colonoscopy of which one patient had a family history of CRC and 8 patients were referred for gastrointestinal symptoms (table 1).

**Table 1:** Demographics of pwCF who underwent colonoscopy

Demographic variables	
Number of pwCF aged ≥30 years who underwent colonoscopy between 2010 and 2020	33
Mean age at colonoscopy (±SD), years	44.8 (±11.0)
Male (%)	23 (70%)
ppFEV_1_ (±SD), %	45.4 (±22.4)%
Phe508del homozygotes (%)	16 (48%)
CFTR modulator treatment (%)	11 (33%)

CFTR, CF transmembrane conductance regulator; FEV_1_, forced expiratory volume in one second; pp, percent predicted; pwCF, patients with cystic fibrosis.

Colonoscopy completion rate was 94.9%. Patients received either standard preparation, which was Moviprep two sachets, one the day before and one on the day of procedure, or an extended bowel preparation regimen with three sachets of MoviPrep. Eighteen patients received standard bowel prep and 12 received the extended preparation. Slightly better diagnostic views were achieved with extended preparation 12 (86%)/14 compared with standard preparation in 18 (72%)/25, however this was not significantly different (p=0.44). In our cohort, bowel prep was rated as ‘good’ in 13 (33%)/39, ‘satisfactory’ in 17 (44%)/39 and ‘inadequate’ in 9 (23%)/39 colonoscopies. Of those with extended bowel prep 83% (10/12) had a BBPS of 5 and above.

**Figure 1:**
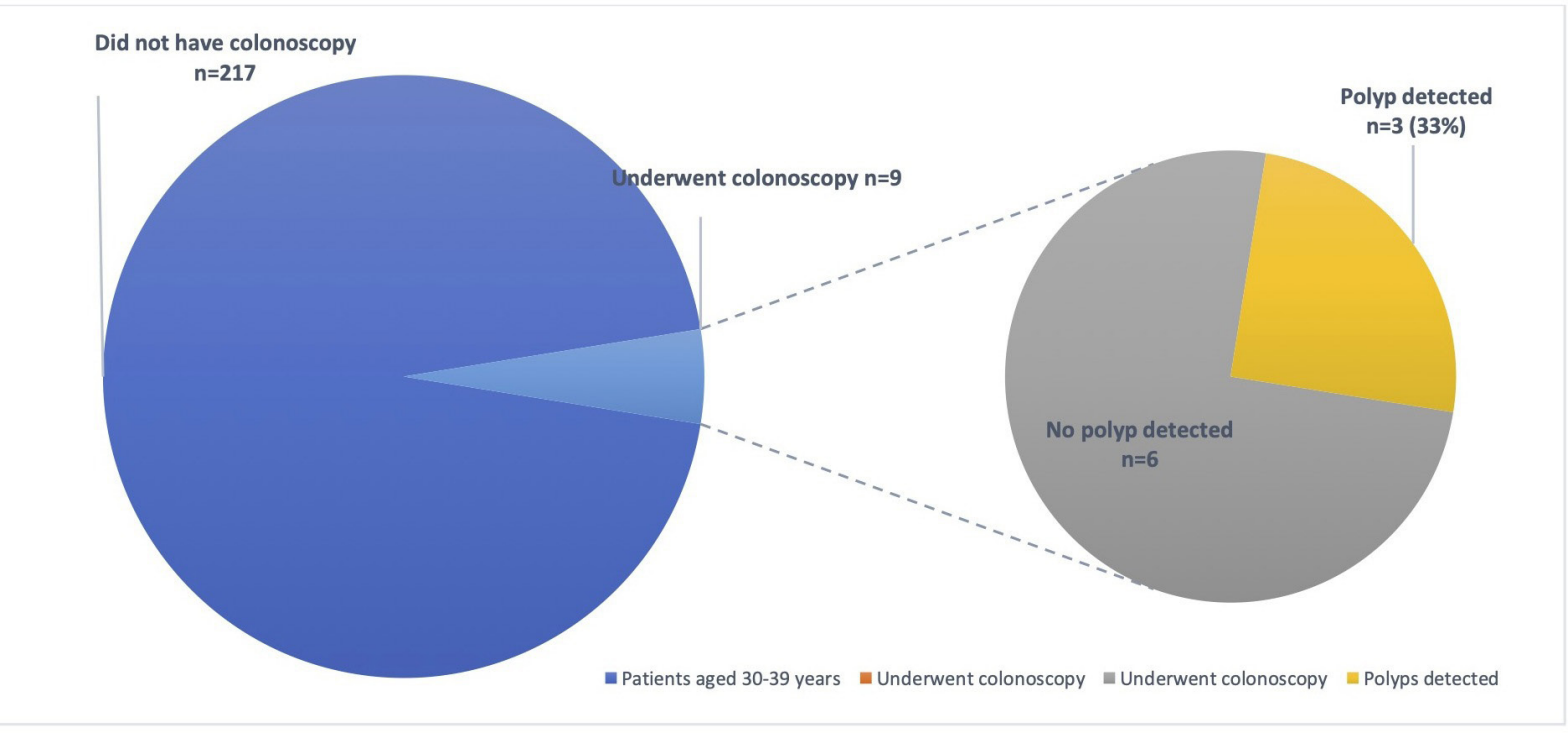
Polyp detection rate in patients with cystic fibrosis aged 30–39 years.

**Figure 2:**
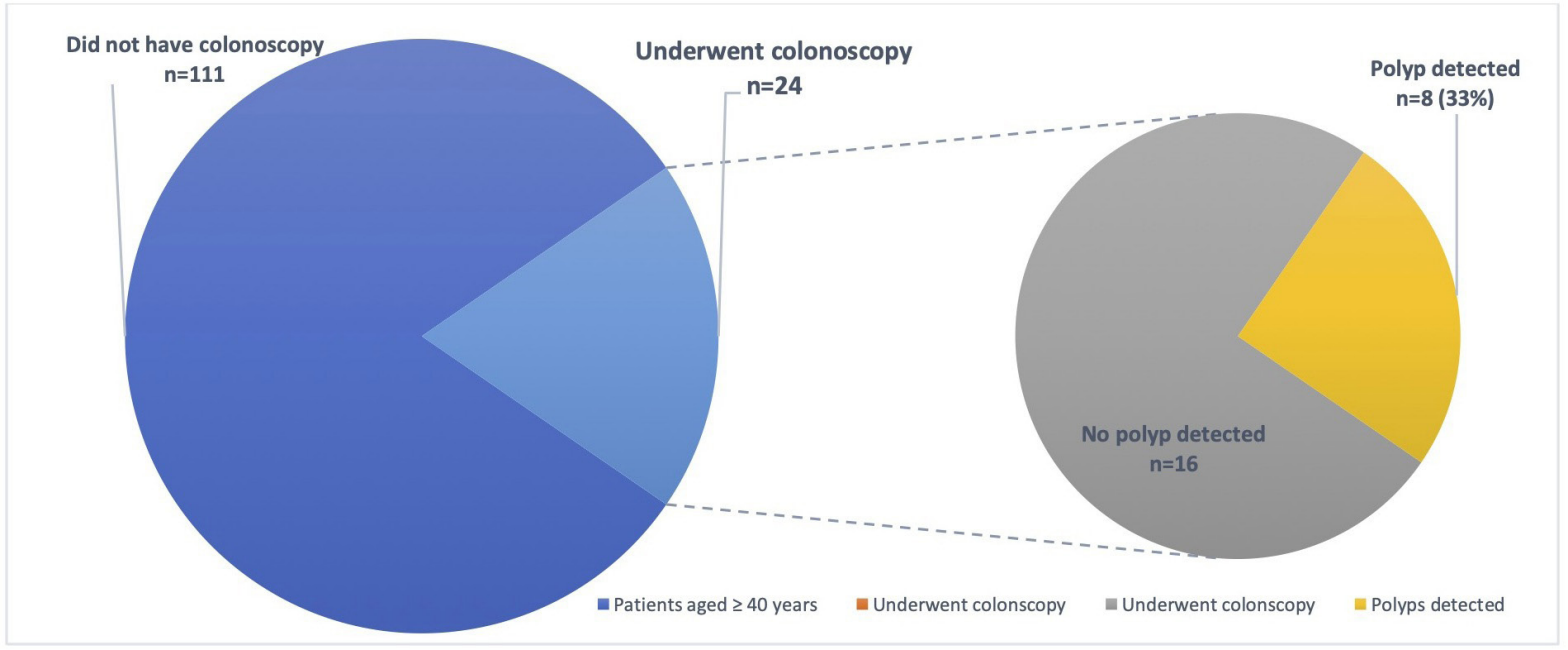
Polyp detection rate in patients with cystic fibrosis aged ≥40 years.

At colonoscopy, in 11 (33%)/33 pwCF, 20 polyps were detected and removed (table 2). Overall polyp retrieval rate was 80%. Subgroup analysis of pwCF aged 30–39 years old versus those aged 40 years and older found the polyp detection rate was similar in both groups. In patients aged 30–39 years, nine patients underwent colonoscopy, of which three (33%) patients had polyps detected and all were found to be premalignant on histology. Of those aged ≥40 years, 24 patients went on to have a colonoscopy, of which eight (33%) patients had polyps detected, with 93.8% confirmed premalignant polyps and 87.5% proven adenomas on histology (figures 1 and 2).

**Table 2:** Polyp size, location and histology in pwCF

	Number of polyps
**Total number of polyps detected between 2010 and 2020 in 33 pwCF aged ≥30 years who had a colonoscopy**	20
Polyp size	
<10 mm	15 (75%)
≥10 mm	5 (25%)
Polyp location	
Left colon (sigmoid or descending)	7 (35%)
Transverse colon	3 (15%)
Right colon (ascending or caecum)	9 (45%)
Rectum	1 (5%)
Polyp histology	
Tubular adenoma with low-grade dysplasia	14 (70%)
Serrated polyp	1 (5%)
Postinflammatory polyp	1 (5%)
Not retrieved for histological analysis	4 (20%)

pwCF, patients with cystic fibrosis.

Over 10 years of follow-up, no patients developed postcolonoscopy CRC. However, of the 111 pwCF aged 40 years and above who did not have a colonoscopy, four (3.6%) patients subsequently developed CRC, three of whom died from complications of their CRC. The pwCF who survived had advanced presentation of rectosigmoid cancer detected on CT abdomen and, subsequent colonoscopy requiring multiple surgeries, is now in remission 3 years on from the initial surgery and is awaiting colostomy reversal.

## Discussion

Recent studies suggest that more than half of the pwCF will develop adenomas by the age of 40 years and a significant proportion of these patients will go onto develop aggressive adenomas and adenocarcinomas.^4^ The yield of premalignant polyps and proven adenomas was high in this cohort however, only 9% of our population with CF aged ≥30 years had a colonoscopy within a 10-year period. During our study, we found that none of the patients who underwent a colonoscopy developed CRC; however, of those patients who did not have a colonoscopy we identified four patients who were diagnosed with CRC, giving a missed cancer rate of 3.6% in this population, which may have been prevented had patients undergone colonoscopy.

Our study also demonstrated that colonoscopy is a well-tolerated procedure, with good completion rates among pwCF, with a high yield of colonic polyps. Despite this, colonoscopies for symptomatic indications were performed infrequently in pwCF at our centre. At present, there are no formal CRC screening guidelines in the UK for CF. There are no randomised control trials comparing outcomes for pwCF who have been screened for bowel cancer against those who have not. The Cystic Fibrosis Colorectal Cancer Screening Task Force in the USA released a recommendation statement in 2018 recognising the increasing life expectancy and the potential aggregated age-related increased risk of CRC in pwCF.^4^ Based on limited available evidence at the time it was suggested the average age of onset of CRC in pwCF is 40 years which is approximately 20–30 years younger than in the population without CF. Interestingly, in our study we found premalignant polyps were identified in even younger patients aged 30–39 years at the same rate as those aged 40 years and older (33%, figures 1 and 2).

For pwCF potential CRC risk factors are present from an early age including high-fat, low-fibre diets, malabsorption of nutrients and chronic intestinal inflammation which may all increase the risk of tumour initiation and colonic adenomas or CRC.^9^ CFTR-knockout mice have shown an increased incidence of colon cancer compared with wild-type mice, and dysregulation of genes associated with immune responses and intestinal stem cells regulation.^7^ Other important factors that can contribute to the increased risk of CRC are having a family history of CRC, gender, age of CF diagnosis, history of meconium ileus or distal intestinal obstruction syndrome, diabetes mellitus and exocrine pancreatic insufficiency.^10^ Frequent exposure to radiation with X-rays and CT scans may also potentially contribute to the increased risk of cancer in pwCF.^9 11^ High-grade dysplasia is found in 25% of all polyps detected by surveillance colonoscopy if performed at 1–2-year intervals in the general population. In pwCF, high-grade dysplasia has been reported to be as high as 50% for adenomatous polyps and 25% for advanced adenomas^12^ providing further data to support a specific screening programme is required for the CF population. Assessing availability of resources and effective risk stratification of pwCF will be important to prioritise those at highest risk without overwhelming endoscopy units. At our CF centre, 135 patients aged ≥40 years would have qualified for CRC screening colonoscopy if they lived in North America. A key finding from our study is that only 17.8% of those who would have been eligible for CRC screening in North America, underwent colonoscopy within a 10-year period at our centre, in the absence of screening guidelines, and in those who did not have a colonoscopy the potentially preventable CRC rate was almost 4%.

Colonoscopy is the gold standard investigation for assessment of CRC, therefore careful service planning and close liaison between CF and endoscopy units will be essential for such screening programmes to be effective in the future. However, more economical, less-invasive and time-consuming tests may be considered to prioritise patients at higher risk. Faecal-immunochemical test (FIT) has been shown to have high sensitivity for the detection of CRC in the general population and in a symptomatic population it can identify those patients who require colorectal investigation with the highest priority.^13^ In 2020, the national screening committee in the UK recommended screening for FIT to be used as a precursor to colonoscopy, as it is an objective measure of risk with a vastly superior positive predictive value compared with symptoms alone for CRC, while also identifying a truly low risk cohort of patients.^13^ It is considered to be as effective in reducing deaths from bowel cancer and to help prevent it.^14^ FIT tests have not as yet been validated in the population with CF. No studies to date have combined and compared the predictivity of FIT screening vers colonoscopy screening for CRC in pwCF.

An alternative non-invasive screening option is the stool DNA test in which stool is assayed for human DNA shed in the colon. Colonic lesions such as adenomatous and serrated polyps contain altered DNA that may be detected by sensitive assays that target specific genetic and epigenetic biomarkers to discriminate neoplastic lesions from non-neoplastic tissue.^15^ Faecal DNA tests have shown slightly reduced specificity compared with FIT tests^15^ and have never been evaluated in pwCF. This test is currently not used for screening purposes in the UK and is considered second tier at present for CRC screening in the US.

Once a patient has been referred for colonoscopy the next important consideration is which bowel preparation regimen is used. In view of their physicochemical characteristics of intestinal mucus and slower intestinal transit time, pwCF are likely to require increased bowel preparation compared with the general population. In our study, patients who received a modified/extended regimen had slightly better diagnostic view rates of 86% versus 72% with standard bowel prep, although this was not found to be statistically significant, likely due to the small numbers of procedures performed. Matson *et al* highlighted there was a significant difference between modified CF bowel preparation group and standard bowel preparation group in bowel visualisation outcomes, with the modified CF bowel preparation group having a higher proportion of ‘excellent’ or ‘good’ colonic visualisation cleanse (50.0% vs 25.9%) and lower rates of ‘poor’ visualisation cleanse (10.5% vs 44.5%) than standard bowel preparation (p=0.006). Adenomatous polyps detection rate at initial colonoscopy was higher in the modified CF bowel preparation cohort than with the standard preparation group (50.0% vs 18.5%, p<0.01).^3^ Colonic adenocarcinoma diagnosis was similar in both groups. Classification and composition of extended bowel prep will vary between centres. The European Society of Gastrointestinal Endoscopy Guideline in 2019 recommended pwCF should have more intensive bowel prep regimens, but no specific laxative or dosage was advised and it suggested additional evidence is needed.^16^ Inadequate bowel clearance for colonoscopy may lead to missed lesions, prolonged procedure duration and repeat procedures. Endoscopists at our centre use the BBPS bowel preparation rating scale as a standardised method of categorising the efficacy of bowel preparation and diagnostic views. In this study, we found the majority (77%) of patients had adequate or good bowel prep. Although BBPS is one measure of efficacy of bowel preparation, reflecting the colon’s cleanliness during the inspection phase of the procedure, other validated bowel preparation evaluation scales exist, involving relatively complex scoring systems.^17 18^ Despite the subjectivity and lack of standardised definition for the terms used in these scales the Bowel Cancer Screening Programme and UK Key Performance Indicators & Quality Assurance Standards for Colonoscopy Report recommends a 4-point scale with terminologies, such as, excellent, adequate, complete despite poor preparation or failed due to poor preparation, should be recorded for all colonoscopies.^17^

The era of CFTR modulator treatment has revolutionised CF management. Survival is likely to increase further following the introduction of highly effective CFTR modulators, which target the underlying protein defect, resulting in clinical improvement and stability. Trials related to CFTR modulators primarily focus on endpoints relating to pulmonary manifestations, however, the impact of these therapies on non-pulmonary manifestations of CF such as gastrointestinal involvement requires equal clinical focus. Partial correction of CFTR may prove to be advantageous in reducing the risk of CRC in the longer term; however, there is an increased risk of CRC associated with an ageing population. Birch *et al* demonstrated than not only are pwCF at increased risk of CRC but the risk is higher with certain CFTR mutations.^19^ Further work is required to establish the association between CFTR mutations and cancer risk.

Mainz *et al* demonstrated that therapy with Elexacaftor/Tezacaftor/Ivacaftor led to a significant reduction in abdominal symptomatology, independent from previous treatment with other CFTR modulators.^20^ Therapy with an earlier generation single agent modulator (Ivacaftor) has shown to improve nutritional status, reduce intestinal inflammation and reduce chronic abdominal symptoms in pwCF carrying at least one gating CFTR mutation.^21 22^ One of the potential mechanisms for improving gut inflammation was the impact on colonic microbiota, including an increase in *Akkermansia* and a reduction in Enterobacteriaceae.^5 21^ Other studies have shown other CFTR modulator combinations to have potent anti-inflammatory properties, in addition to their ability to stimulate CFTR function, which could contribute to better clinical outcomes.^23^ However, ultimately the full implications of CFTR modulation on carcinogenesis are yet to be fully established and are likely to evolve with the introduction of newer agents. Furthermore, there still remains a proportion of the CF population with rarer mutations that are not as yet eligible for any modulator therapy.

### Limitations

This is a retrospective a single-centre study with a small sample size, limiting its statistical power. It is unclear whether our data are representative of the national picture, and practices in other centres in the UK. Although colonoscopy was effective and readily available at our CF centre, the utility and accessibility to colonoscopy may vary between centres. Multicentre evaluation of the utility, diagnostic yield and availability of colonoscopy from other CF centres in the UK would therefore be helpful going forward. The bowel preparation data in this ‘real-world’ experience will be helpful in future prospective studies. Even with our extended bowel preparation regime, the diagnostic view rates of 86% are still below the standards required for CRC screening programmes, and therefore it is likely that pwCF need a more customised and enhanced bowel preparation regime, and this should be determined in future studies. Moreover, in the context of suboptimal bowel preparation in 23% of pwCF who had colonoscopy, we cannot exclude that some further polyps may have been missed and therefore the polyp detection rate could have been even higher than what we have reported.

## Conclusion

In this series, where the utility of colonoscopy for symptomatic indications in pwCF was very low, the data highlight the risk of potentially preventable CRC in those ≥40 years. In the minority who had a colonoscopy, the yield for premalignant polyps was high with no cases of postcolonoscopy CRC, and a 3.6% incidence of CRC in those >40 years who had not been investigated. CF clinicians and gastroenterologists in CF centres should be vigilant of the risks of CRC, which are likely to become even higher with increasing life expectancy.

## Data Availability

Data are available upon reasonable request. Not applicable.
